# Cumulative Risk as a Marker of Social Context

**DOI:** 10.1002/cbm.2378

**Published:** 2025-03-28

**Authors:** Kyle Treiber, Per‐Olof H. Wikström

**Affiliations:** ^1^ Institute of Criminology University of Cambridge Cambridge UK; ^2^ Malmö University Malmö Sweden

**Keywords:** cumulative risk, PADS+, SAT, situational action theory, social context

## Abstract

**Background:**

This paper takes on ‘*Farrington's challenge*’ ‘to bridge the gap between risk factor research and more complex explanatory theories’ by offering an explanation for the unexplained statistical phenomenon of cumulative risk. We argue that cumulative risk primarily reflects the social contexts which crime relevant causal processes operate in and draw upon for their content and efficacy.

**Aims:**

This paper tests if the immediate causes of crime according to Situational Action Theory (crime propensity and criminogenic exposure) can account for the relationship between cumulative risk (reflecting key features of family, neighbourhood, school and peer contexts) and crime involvement.

**Methods:**

The paper uses data from the Peterborough Adolescent and Young Adult Development Study (PADS+) reflecting the social lives and criminal behaviour of a randomly sampled UK age cohort from ages 12 to 24 (2003–2016). Data used are drawn from parent and participant questionnaires, space–time budgets, community surveys, the UK Census and land use databases.

**Results:**

Cumulative risk statistically accounts for 7% and 8% of the variance in crime prevalence and frequency, respectively, whereas crime propensity and criminogenic exposure account for 52% and 58%, respectively. Moreover, and importantly, measures of crime propensity and criminogenic exposure fully account for (statistically mediate) the association between cumulative risk and both crime prevalence and crime frequency.

**Conclusions:**

Cumulative risk does not represent cumulative causation. The phenomenon of cumulative risk is best understood as representing the social context. Future research should focus on identifying features of social contexts that provide relevant content to and impact the efficacy of key action (and developmental) processes in crime causation.

## 1 Introduction

In this brief paper we will take on ‘*Farrington's challenge*’ ‘to bridge the gap between risk factor research and more complex explanatory theories’ (Farrington 2000, 7). We will focus on the phenomenon of ‘cumulative risk’ for crime—the finding that the more risk factors (statistically significant predictors) for crime people exhibit, the more likely they are to also display greater crime involvement (e.g., Loeber, Slot, and Stouthamer‐Loeber 2006, 161). Cumulative risk (CR) has recently been lauded as ‘the most salient way to approach the study of the causes of criminal behaviour’ (TenEyck, Barnes, and El Sayed 2023, 556). However, this suggestion is undermined by the fact that, as Evans, Li, and Whipple (2013, 1,380) highlighted more than a decade ago, ‘one of the primary limitations of CR is the lack of theoretical explanation for its predictive power’. It is certainly challenging to explain why ‘the content generally seems less important than the number of risk factors’ (Andershed, Gibson, and Andershed 2016, 78). Some scholars have concluded (we suggest prematurely) that ‘the most pragmatic solution is to simplify the chaotic nature of human behaviour into patterns that may occur on average’ (TenEyck, Barnes, and El Sayed 2023, 561–562).

We argue that the risk factor approach is flawed not only because it concedes to a limited understanding of *why* crime events happen and *why* criminal careers unfold as they do but also, and consequently, because it provides a limited understanding of *how* to effectively prevent crime events from happening and criminal careers from unfolding. As Farrington (2000, 7) has highlighted, ‘a major problem of the risk factor prevention paradigm is to determine which risk factors are causes and which are merely markers or correlated with causes’. By focusing on statistical relationships and predictors, the risk focused approach detracts attention from causal processes that link factors to crime events and criminal careers and should be the target of crime prevention efforts.

Rather than a risk focused approach that attempts to account for the complex nature of criminal behaviour through a collection of commonly poorly specified and unreliable probabilities, we advocate a theory‐driven approach that foregrounds causal processes rather than statistical relationships to provide a framework for mapping out how factors are associated with crime, for example, through causal relevance (direct or indirect), or through selection effects which position them as markers for factors that are causally relevant (Wikström 2006; Wikström and Kroneberg 2022; Wikström and Treiber 2013). In this paper, we offer a *theoretical* explanation for the link between cumulative risk and crime involvement and provide a preliminary *empirical* illustration of this approach and how it can help identify causally relevant factors that explain, and account for, the relationship between CR and long‐term patterns of crime involvement, and how it can present a more productive focus for the advancement of crime prevention policy and practise.

## 2 Cumulative Risk Does Not Equal Cumulative Causation

We suggest that rather than cumulative causation, a more plausible explanation for the statistical phenomenon of cumulative risk is ‘that many established risk factors (predictors) are markers of qualities of the various life domains (family, school, work, leisure, etc.) that constitute the social context in which the [crime] relevant causal processes affecting a person operate and on which they depend for their *content* and *efficacy*’ (Wikström, Treiber, and Roman 2024, 15–16). The inclusion of multiple markers in a predictive model may strengthen the model's *representation of the social context* in which a person acts and develops, for example, the circumstances in which a person encounters particular (crime‐relevant) motivators, the prevailing cultural (e.g., socio‐moral) milieu, and the social structural features that create intersections between particular people (with particular crime propensities) and settings (with particular criminogenic inducements) (Wikström, Treiber, and Roman 2024, 109).

To understand the role of these factors in crime causation we need a theory that models the causal processes that link them to crime. We draw upon Situational Action Theory (SAT) as it specifies (1) the situational processes that link people to acts of crime and the personal and environmental factors that directly cause crime, and (2) the developmental processes that shape the emergence and convergence of those causal factors over time (Wikström, Treiber, and Roman 2024, 29–112). Together these mechanisms provide a theoretical framework for understanding the role of social contexts in crime and, we suggest, also the phenomenon of cumulative risk.

## 3 Situational Action Theory (SAT)

SAT asserts that people are the source of their actions and that their actions are essentially rule guided (Wikström 2006, 2010). On this basis, the theory asks questions about what makes people perceive and choose crime as an action alternative in response to specific motivations they encounter in various circumstances, and what personal and environmental factors are directly (as causes) and indirectly (as causes of the causes) causally important in that process. SAT suggests that the key causally effective *inputs* to the perception–choice process are (1) people's crime propensity (their tendency to see and choose crime as an action alternative), based on their personal morality (their moral values/rules and related emotions) and their ability to exercise self‐control (their ability to act in accordance with their morality when externally challenged) and (2) the criminogeneity of the setting in which they take part (its tendency to normalise or encourage acts of rule breaking), based on the moral norms of the setting and their level of enforcements (i.e., the setting's capacity to make people refrain from acts of rule breaking they consider). SAT further suggests that (1) people's crime propensity is largely a result of psychoecological processes of moral education (affecting personal morality) and cognitive nurturing (affecting the ability to exercise self‐control), and (2) their criminogenic exposure is largely a result of socioecological processes of social‐ and self‐selection (see e.g., Wikström, Treiber, and Roman 2024, 64–112, for more details of these processes). Crucial for our discussion of cumulative risk as a marker of social context is SAT's assertion that the key causal processes it identifies all take place in and are dependent on their social context for their content and efficacy.

## 4 Cumulative Risk as a Marker of Social Context

Frequently studied family, neighbourhood, school and peer risk factors (predictors) in criminology are typically factors that do not move people to action (e.g., to commit acts of crime). No one commits an act of crime solely because their parents have a low income, they have a poor relationship with their parents and teachers or they live in a disadvantaged neighbourhood. Although studies generally find it is more common that young people with these kinds of characteristics (or backgrounds) are involved in frequent and serious crime, most young people with these characteristics do not engage in criminal activities more often than young people in general. In other words, there is commonly large unexplained within‐group variation in crime involvement among those who share an identified ‘risk factor’ or even combinations of risk factors.

Many kinds of risk factors may rather be seen as indicators of the social context of people's actions, reflecting qualities of key life domains in which they perceive and choose their options for action; many may also be indicators of the social context of people's development, reflecting qualities of key life domains in which they acquire their crime propensities (are morally educated and cognitively nurtured) and are exposed to criminogenic settings (through processes of selection). Qualities of these key life domains consequently predict crime involvement because they are associated with contexts in which people may situationally encounter crime‐relevant motivators, weak moral contexts and a lack of effective controls; or developmentally experience less penetrating law‐relevant moral education, less intensive cognitive nurturing or greater constraints on their access to settings with fewer criminogenic inducements. Importantly, as markers for these social contexts, risk factors do not exhibit any causal efficacy.

For example, having many children in a household may make it more difficult for some parents to provide effective moral education and cognitive nurturing, or to constrain their children's activity fields to limit their access to criminogenic settings. But this is far from inevitable, especially in social contexts where the culture and social structure is able to support large families. Consequently, it is not the size of the family in itself that is criminogenic; nor is family size an appropriate reason or target for intervention. What is causally relevant, and would be an important target for intervention, are experiences of moral education, cognitive nurturing and selection, and the crime propensities and criminogenic exposure they generate. In other words, it is not the markers of context that are important but rather what they indicate about the content and efficacy of key causal processes of action and development within those contexts.

## 5 Data and Methods

The data used in this analysis are drawn from the Peterborough Adolescent and Young Adult Development Study (PADS+), an ongoing longitudinal study of a random population sample of 716^1^ participants born between September 1 1995 and August 31 1996 who grew up in the UK city of Peterborough (Wikström, Oberwittler, et al. 2012; Wikström, Treiber, and Roman 2024). Interviews were conducted with participants' parents in 2003, the year participants turned 12, to collect contemporary and retrospective data on their childhood experiences. Participants were then interviewed annually from ages 13 to 17, and at ages 19, 21 and 24. During these interviews data were collected through multiple methods including questionnaires, cognitive tests, event calendars and space–time budgets. These interview data were linked to external data including the UK Census data, two project‐linked small‐area Peterborough Community Surveys (PCS) in 2005 and 2012^2^ (Oberwittler and Wikström 2009; Wikström, Treiber, and Hardie 2012) and criminal justice records. In this paper we utilise data from the parent and participant questionnaires, the space–time budget, the PCS, the 2001 UK Census and land use databases for the 615 participants (87%) who took part at all ages.^3^ For further details regarding sampling, response rates and methods used see (Wikström, Treiber, and Roman 2024, 115–177; Wikström, Oberwittler, et al. 2012, 44–106).

### 5.1 Crime Variables

For *crime prevalence and frequency* we utilise a measure of self‐reported crime from participants' questionnaires.^4^ In each interview, participants were asked if they had committed arson, vandalism, shoplifting, theft from a person, assault, robbery, residential burglary, nonresidential burglary or car crime (theft of or from a car) in the past year (prevalence), and if so how many times (frequency). Participants with a crime prevalence reported at least one of these crime types between ages 13 and 24. Their crime frequency was calculated as the number of these crimes they reported from ages 13 to 24. Of the 615 participants, 445 (72.4%) reported at least one such act during the study period, and altogether they reported a total of 17,559 crimes (Max = 1937, Mean = 28.6, Mdn = 4).

### 5.2 Risk Domain Variables

All *risk factor predictors* reflect conditions during childhood (pre‐adolescence) and were measured through parent interviews in the year participants turned 12, or characterised participants' home output areas at the time of the parent interviews drawing on data from the 2001 UK Census and the PCS 2005. Predictors are operationalised dichotomously such that 1 indicates being ‘at risk’ and 0 indicates ‘not at risk’. Predictors were selected to reflect four key life domains—family, neighbourhood, school and peers—during participants' childhood (Table 1). Three factors were selected for each domain reflecting their resources, relationships and functioning. Details on the specific content and coding of included risk domain variables are provided in Appendix A.

**TABLE 1:** Operationalisation of ‘at risk’ for key predictors.

Domain	Characteristic	Predictor	‘At risk’	% Of sample at risk
Family	Resources	Family social position	1 SD below the mean	18.4
	Relationships	Family cohesion	1 SD below the mean	19.2
	Functioning	Parental knowledge	1 SD below the mean	19.6
		Family domain risk indicator	Any risk in family domain	43.5
Neighbourhood	Resources	Neighbourhood social disadvantage	1 SD above the mean	19.0
	Relationships	Neighbourhood social cohesion	Lowest 25%	25.1
	Functioning	Neighbourhood informal social control	Lowest 25%	25.6
		Neighbourhood domain risk indicator	Any risk in neighbourhood domain	38.1
School	Resources	Parents' opinion of the school	Poor/very poor opinion	5.9
	Relationships	Child's relationship with teachers	Does not get on well with teachers	4.7
	Functioning	Child's opinions of school	Does not like school	10.6
		School domain risk indicator	Any risk in school domain	15.6
Peers	Resources	Time with friends	Most or all days	30.4
	Relationships	Parent approval of peers	Ambivalence or disapproval	12.2
	Functioning	Peer crime and drug use	Some/most friends involved in crime or drug use	4.4
		Peer domain risk indicator	Any risk in peer domain	41.4

### 5.3 Cumulative Risk Scores

There are many different ways to operationalise risk and quantify cumulative risk (see Evans, Li, and Whipple (2013) for a detailed review); overall, critical and empirical assessment indicates that a CR score as a sum of types of risk provides comparable or in some cases superior predictive power, especially developmentally (Burchinal et al. 2000; Evans, Li, and Whipple 2013), and has a number of advantages over other approaches (e.g., in regards to parsimony, statistical power, multicollinearity, weighting and measurement error).

A *total cumulative risk score* was created to indicate how many of the 12 risk indicators the participant exhibited (risk variety score). This indicator will be used to assess if the predictive ability of cumulative risk is accounted for (statistically mediated by) crime propensity and criminogenic exposure. For each domain (family, neighbourhood, school and peers) a *domain risk score* was also created by summing individual domain risk indicators.

### 5.4 Explanatory Variables^5^ ^,^ ^6^

*Crime propensity* is measured by standardising and summing two scales from participants' questionnaires: *personal moral rule*s and *generalised ability to exercise self‐*control. We use a sum of crime propensity scores across all ages; higher scores reflect higher crime propensities.

Criminogenic exposure is a complex measure utilising data on time use, socioecological factors and personal experiences drawn from space–time budgets, community surveys and participant questionnaires. It is measured by standardising and summing two measures: *time spent in criminogenic settings* and *crime prone peers*. As with crime propensity, we use a sum of criminogenic exposure scores across all ages; higher scores reflect higher criminogenic exposure.

## 6 Analytic Strategy

**FIGURE 1:**
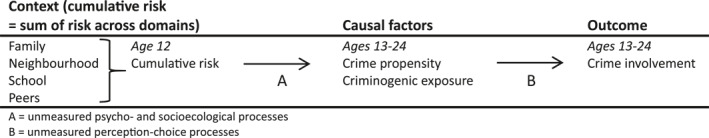
The influence of childhood social context (cumulative risk) on young people's crime involvement accounted for by its influence on their crime propensity and criminogenic exposure.

Based on our theoretical assumptions we hypothesise that the statistical association between childhood cumulative risk (as a marker of childhood context) and subsequent crime involvement can be accounted for (statistically mediated by) its influence on participants' crime propensity and criminogenic exposure as illustrated in Figure 1. Our analyses are presented in three steps.

1. We assess the relationship between cumulative risk and crime prevalence (using *Χ* ^2^ tests) and crime frequency (using Mann–Whitney tests).
2. We assess the relationship between cumulative risk and proposed explanatory variables crime propensity and criminogenic exposure (using OLS regression).
3. We assess if the proposed explanatory variables fully statistically account for the relationship between cumulative risk and crime prevalence (using logistic regression) and crime frequency (using multiple OLS regression).

## 7 Findings

### 7.1 Relationship Between Risk Scores and Crime Involvement

#### 7.1.1 Risk Domain Indicators

A slightly higher proportion of those at risk offended than those not at risk for each risk domain (Table 2), and those at risk offended more frequently (Table 3). However, the effect sizes (odds ratios, *r* values) show that the predictive power of domain risk indicators are rather small.

**TABLE 2:** *Χ* ^2^ tests for whether those at risk were significantly more likely to report offending (have a crime prevalence) between ages 13 and 24.

Domain risk indicators	% Offenders		*Χ* ^2^			Odds ratios
	No risk	At risk	*Χ* ^2^	Sig	Phi	Risk versus no risk
Family domain risk indicator	65.3	81.6	19.894	0.000	0.180	1.25
Neighbourhood domain risk indicator	69.4	78.8	6.139	0.013	0.103	1.14
School domain risk indicator	69.9	86.5	11.153	0.000	0.135	1.24
Peer domain risk indicator	67.1	79.4	10.743	0.001	0.134	1.18

**TABLE 3:** Mann–Whitney tests for mean differences in crime frequency between ages 13 and 24 for those at risk and not at risk.

Domain risk indicators	Crime frequency (ages 13–24)					
	Mean		Mann–Whitney			
	No risk	Risk	*U*	*p*	*Z*	*r*
Family domain risk indicator	24.37	34.25	56,450.5	0.000	4.9	0.20
Neighbourhood domain risk indicator	19.02	46.99	46,850.5	0.000	3.5	0.15
School domain risk indicator	21.43	67.28	30,585.0	0.000	3.6	0.15
Peer domain risk indicator	20.70	32.83	52,249.5	0.000	4.4	0.18

#### 7.1.2 Cumulative Risk Scores

As expected, the cumulative risk score across all risk indicators across all domains predicted crime prevalence (Table 6) and crime frequency (Table 7). However, the effect sizes are modest (e.g., for prevalence Nagelkerke *R* ^2^ × 100 = 7.6%, whereas for crime frequency *R* ^2^ × 100 = 7.1%).

#### 7.1.3 Relationship Between Risk Scores and Proposed Explanatory Variables

All risk domains were associated with higher mean crime propensity and criminogenic exposure (Table 4). The *cumulative risk score* predicted crime propensity and criminogenic exposure (*p* < 0.001, Table 5), although the amount of variance explained is rather small (*R* ^2^ × 100 = 9.8% for crime propensity and 11.4% for criminogenic exposure). This suggests cumulative risk is not only a rather weak predictor of crime involvement but also a rather modest proxy for people's crime propensity or criminogenic exposure.

**TABLE 4:** Mann–Whitney tests for mean differences in crime propensity and criminogenic exposure between ages 13 and 24 for those at risk and not at risk.

Domain risk indicators	Crime propensity						Criminogenic exposure					
	Mean		Mann–Whitney				Mean		Mann–Whitney			
	No risk	Risk	*U*	*p*	*Z*	*r*	No risk	Risk	*U*	*p*	*Z*	*r*
Family domain risk indicator	−1.95	2.07	55,108.0	0.000	4.7	0.19	−1.47	2.06	53,219.0	0.000	4.8	0.20
Neighbourhood domain risk indicator	−0.99	1.53	44,582.0	0.004	2.9	0.12	−0.82	2.09	44,312.0	0.000	3.6	0.15
School domain risk indicator	−1.34	5.68	33,373.0	0.000	5.9	0.24	−0.80	4.80	31,104.0	0.000	5.5	0.23
Peer domain risk indicator	−1.91	2.01	52,016.0	0.000	4.7	0.19	−1.60	2.39	51,542.0	0.000	5.4	0.22

**TABLE 5:** Regressions for cumulative risk scores predicting crime propensity and criminogenic exposure.

Risk score	Crime propensity					Criminogenic exposure				
	*B*	SE	*β*	*p*	*R* ^2^ × 100	*B*	SE	*β*	*p*	*R* ^2^ × 100
Total cumulative risk	1.680	0.215	0.315	0.000	9.8%	1.529	0.182	0.340	0.000	11.4%
(Constant)	−3.390	0.594		0.000		−2.700	0.503		0.000	

### 7.2 Explanatory Variables and Statistical Mediation

#### 7.2.1 Crime Prevalence

Table 6 shows that crime propensity and criminogenic exposure (model 2) fully statistically mediate the relationship between crime prevalence and the cumulative risk scores (model 1). The inclusion of crime propensity and criminogenic exposure dramatically increases the effect size, from Nagelkerke *R* ^2^ × 100 = 7.6% to Nagelkerke *R* ^2^ × 100 = 52.5%.

**TABLE 6:** Total childhood cumulative risk score predicting crime prevalence from ages 13 to 24.

	Model 1						Model 2					
	*B*	SE	*p*	Exp(*B*)	Low CI	Upper CI	*B*	SE	*p*	Exp(*B*)	Low CI	Upper CI
(Constant)	0.440	0.238	0.001	1.553			2.039	0.260	0.000	7.686		
Total cumulative risk score	0.320	0.066	0.000	1.377	1.210	1.568	0.084	0.082	0.305	1.087	0.927	1.276
Crime propensity							0.087	0.019	0.000	1.091	1.052	1.132
Criminogenic exposure							0.266	0.037	0.000	1.304	1.213	1.403

	−2LL	Block sig	Cox and Snell	Nagelkerke *R* ^2^ × 100			−2LL	Block sig	Cox and Snell	Nagelkerke *R* ^2^ × 100		
	603.262	0.000	0.052	7.6%			389.674	0.000	0.362	52.5%		

#### 7.2.2 Crime Frequency

Table 7 shows that crime propensity and criminogenic exposure also fully statistically mediate the relationship between cumulative risk and crime frequency. The total cumulative risk score significantly predicts crime frequency but accounts for only 7.1% of the variance (model 1). Once crime propensity and criminogenic exposure are entered into the model, cumulative risk is no longer significantly associated with crime frequency, and 58.4% of the variance is explained (model 2).

**TABLE 7:** Total childhood cumulative risk score predicting crime frequency from ages 13 to 24.

	Model 1				Model 2			
	*B*	SE	*β*	*p*	*B*	SE	*β*	*p*
(Constant)	0.624	0.042		0.000	0.811	0.029		0.000
Total cumulative risk score	0.098	0.015	0.269	0.000	−0.001	0.011	−0.002	0.948
Crime propensity					0.028	0.003	0.411	0.000
Criminogenic exposure					0.034	0.003	0.422	0.000
*R* ^2^	7.1%				58.4%			

## 8 Discussion

In this preliminary analysis, we have found consistent support for the proposition that cumulative risk captures at best factors associated with the causes of crime, and that crime propensity and criminogenic exposure are more directly associated with crime involvement. We have suggested that the increasing predictive power of cumulative risk occurs because many risk factors are associated with social contexts conducive to the development or expression of crime propensities, and the emergence of and exposure to criminogenic contexts. We consequently argue that crime propensity and criminogenic exposure, and the processes through which they emerge, are more credible targets for effective policy and prevention than risk factors and cumulative risk.

We have presented a causal model as an alternative to a probabilistic model to explain the relationship between cumulative risk and crime. We have suggested that many risk factors for crime capture crime‐relevant features of social contexts—that is, features associated with relevant content for, or the efficacy of processes that shape the development of crime propensities (moral education and cognitive nurturing), the emergence of criminogenic inducements (moral norm formation and enforcement) and the exposure of those with high crime propensities to criminogenic settings (self and social selection). We have suggested that cumulative risk increasingly predicts crime because with each additional risk factor the scale more closely reflects the most criminogenic social contexts—those in which people develop the highest crime propensities and are exposed most frequently to the most criminogenic settings and circumstances.

Consistent with Appleyard et al. (2005, 243) we suggest that ‘once we better understand [the mediators and moderators of resilient or adaptive outcomes for children in high‐risk settings]… we will be able to design more effective prevention and intervention programs’. Situational action theory frames precisely these ‘mediators and moderators’, and more importantly, the processes through which they shape crime and crime‐relevant outcomes, and therefore provides a specific guide for the design and development of focused crime prevention efforts. In particular, SAT suggests that the most effective crime prevention will target features of social contexts linked to crime‐relevant content (input) or the efficacy of crime‐relevant processes, that is, of moral education (e.g., antisocial values or inconsistent enforcement of rule following), cognitive nurturing (e.g., reinforcement of impulsive behaviour or factors that reduce executive functioning), social emergence (e.g., cultural ideals upholding particular norms or resource distribution shaping informal social control), and social and self‐selection (e.g., incentives for or impediments to certain kinds of people taking part in certain kinds of settings). Successfully targeting these features of key social contexts (family, school, neighbourhood and peers) can significantly impact crime involvement by reducing people's development of crime propensities and exposure to criminogenic contexts.

## Ethics Statement

This research has been approved by the University of Cambridge Institute of Criminology Ethics Committee and meets the UK Economic and Social Research Council's (ESRC's) requirements for research ethics.

## Conflicts of Interest

The authors declare no conflicts of interest.

## Data Availability

Data sharing is not applicable to this article as no new data were created or analysed in this study.

## Endnotes

^1^
From an initial sampling frame of 2270 young people across the age cohort in the eligible study area, 1000 were randomly sampled, of which 991 were still eligible at the time of data collection. Of these, the parents of 716 (72.3%) agreed to take part.

^2^
The 2005 and 2012 Peterborough Community Surveys involved random samples of residents distributed across all UK Census output areas in the study area. For the PCS 2005, 6615 of the 12,681 randomly sampled residents took part (53.1%, 11.1% of the eligible population). For the PCS 2012, 6839 of the 15,940 randomly sampled residents took part (45.4%, 11.6% of the eligible population).

^3^
PADS+ methodologies ensure very little data (< 1%) is missing from the participants' interviews, including the questionnaires and space–time budgets.

^4^
For a detailed discussion of PADS+'s self‐report crime data, its strengths and limitations, and its comparison with official police records, see Wikström, Treiber, and Roman (2024, 147–177).

^5^
We discuss the reference period for studying the relationship between crime and crime propensity and criminogenic exposure data in Wikström, Oberwittler, et al. (2012, 129–131) and in more detail, incorporating original analyses of contemporaneous criminality using space–time budget data, in Wikström, Treiber, and Roman (2024, 156–162). In this paper we report findings using composite measures of crime, crime propensity and criminogenic exposure across ages 13 to 24. We have also run the same analyses (1) using measures of crime propensity and criminogenic exposure from the same year to predict crime at each age, and (2) using measures of crime propensity and criminogenic exposure from the previous interview (1–3 years before) to predict crime at each age (although we do not recommend lagging crime data this way due to the timing of proposed causal mechanisms). Across all these models, cumulative risk was a much weaker predictor of crime prevalence and frequency than crime propensity and criminogenic exposure, and did not predict crime prevalence or frequency once crime propensity and criminogenic exposure were taken into account.

^6^
For more details on measures of personal morality, ability to exercise self‐control, crime propensity, time spend in criminogenic settings, crime prone peers and criminogenic exposure see Appendix A.

## Appendix A

### Risk Factor Predictors and Their Operationalisation by Domain

All *risk factor predictors* were measured through parent interviews in the year participants turned 12, or characterised participants' home output areas at age 12 drawing on data from the 2001 UK Census and the PCS 2005.

We focus on social contextual factors consistent with our proposition that cumulative risk is associated with features of social contexts. The relationships between the selected risk factors and crime are well known and established in previous research (see e.g., Ellis, Farrington, and Hoskin 2019). A key argument in the cumulative risk literature, which we question in this paper, is that the content of these factors does not matter as much as the number. We argue that causal, not probabilistic, relationships explain these outcomes, and thus we disagree that which factors are included is unimportant. This is why we selected factors to reflect four key social contexts, and key characteristics of those contexts—how well resourced they are, the strength of the participant's relationship to them and how well they function as contexts of action and development—although space constraints do not allow us to analyse the latter in this paper.

#### Family Domain

*Family resources* were characterised by the *family's social position*, measured through a scale of household income, parents' occupational status^7^ and parents' educational attainment.^8^ Higher values indicate higher family social position. Participants scoring one standard deviation below the mean were designated ‘at risk’. *Family relationships* were characterised by a measure of *family cohesion*. This scale included three items: ‘We are a happy family that like each other a lot’; ‘We are a very close family that like to spend time together as much as we can’; ‘We have a lot of conflicts in the family and often get angry with each other’ (reverse‐coded) (five response categories ranging from ‘Strongly disagree’ = 0 to ‘Strongly agree’ = 1). Higher scores indicate greater family cohesion. Participants scoring one standard deviation below the mean were designated ‘at risk’. *Family functioning* was characterised by *parental knowledge* of the child's activities outside the home, using three items: ‘Do you usually know where X is/what X is doing/which friends X is with when out in the afternoon/evening?’ (four response categories ranging from ‘No, never’ = 0 to ‘Yes, always’ = 3). Higher scores indicate greater parental knowledge. Participants scoring one standard deviation below the mean were designated ‘at risk’.

#### Neighbourhood Domain

In PADS+, neighbourhoods were defined by output areas (OAs), the smallest geographic unit available for the UK Census.^9^ These small geographical units closely capture the environment immediately surrounding the participants' homes. *Neighbourhood resources* were characterised by factor scores of *neighbourhood social disadvantage* derived from a principal component analysis using data from the 2001 UK census at the OA level reflecting the percentage of area residents who were working class, had no or low educational qualifications, were unemployed or resided in detached houses (see Wikström, Oberwittler, et al. 2012 for details). Higher scores indicate greater social disadvantage. Participants whose home OA at the time of the parent interview scored one standard deviation above the mean were designated ‘at risk’. *Neighbourhood relationships* were characterised by a measure of *neighbourhood social cohesion* drawing on data from the 2005 Peterborough Community Survey covering five items: ‘People around here are willing to help their neighbours’; ‘This is a close‐knit neighbourhood’; ‘People in this neighbourhood can be trusted’; ‘People in this neighbourhood generally get along with each other’; ‘People in this neighbourhood share the same values’ (four responses categories ranged from ‘Strongly agree’ = 3 to ‘Strongly disagree’ = 0). Scores reflect an empirical Bayes estimate adjusted for individual level socio‐demographic composition. Higher scores reflect higher social cohesion. Participants living in neighbourhoods scoring in the lowest 25% at the time of the parent interviews were designated ‘at risk’. *Neighbourhood functioning* was characterised by a measure of *neighbourhood informal social control* that also draws on data from the 2005 Peterborough Community Survey covering four items: ‘If a group of neighbourhood children were skipping school and hanging out on a street corner, how likely is it that your neighbours would do something about it?’; ‘If some children were spray‐painting on a local building, how likely is it that your neighbours would do something about it?’; ‘If there was a fight in front of your house and someone was being beaten or threatened, how likely is it that your neighbours would break it up?’; ‘If a child was showing disrespect to an adult, how likely is it that neighbours would tell off or scold that child?’ (four responses categories ranged from ‘Very likely’ = 3 to ‘Very unlikely’ = 0). Scores reflect an empirical Bayes estimate adjusted for individual level socio‐demographic composition. Higher scores reflect higher informal social control. Participants living in neighbourhoods scoring in the lowest 25% at the time of the parent interviews were designated ‘at risk’.

#### School Domain

*School resources* were measured through one item asking parents their *opinion of their child's school* (‘What is your opinion about X's school?’ with response categories ‘It is a very poor school’; ‘It is a poor school’; ‘It is a good school’; ‘It is a very good school’). Participants whose parents described their school as poor or very poor were designated ‘at risk’. *School relationships* were characterised by one item asking parents *how well their child generally got along with his/her teachers* (‘Does X generally get along well with most of his/her teachers?’ with response categories ‘No, not at all’; ‘No, not very well’; Yes, quite well’; Yes, very well’). Participants whose parents reported they did not get along very well or at all with their teachers were designated ‘at risk’. *School functioning* was characterised by one item asking parents about their *child's opinion of their school* (‘Does X like to go to school?’ with response categories ‘No, not at all’; ‘No, not very much’; Yes, quite a lot’; ‘Yes, very much’). Participants whose parents reported they do not like school very much or at all were designated ‘at risk’.

#### Peer Domain

*Peer resources* were characterised by *time spent with peers* at home each week (‘How many times per week does X have friends at home?’ with response categories ‘Never’; ‘Some days (1–3)’; ‘Most days (4–6)’; ‘All days (7)’). Participants whose parents reported they spent time with their peer most or all days of the week were designated ‘at risk’. *Peer relationships* were characterised by a measure of *parents' approval of the participant's peers* (‘Do you approve of X's peers?’ with five response categories from ‘Strongly disapprove’ to ‘Strongly approve’). Participants whose parents were ambivalent (‘neither approve nor disapprove’) or disapproved/strongly disapproved of their peers were considered ‘at risk’. *Peer functioning* was characterised by parents' reports of *participants' peers' crime involvement and drug use* (‘Are any of X's friends involved in crime or drug use?’ with response categories ‘No, none of them’; ‘Yes, some of them’; ‘Yes, most of them’). Very few parents reported that any of their child's friends used drugs or were involved in crime; any participants whose parents did were designated ‘at risk’.

### Explanatory Variables and Their Operationalisation

*Crime propensity* is measured using two scales from participants' questionnaires: *personal moral rule*s and *generalised ability to exercise self‐control*. *Personal moral rules* was measured using a 16‐item scale asking participants ‘How wrong is it for someone of your age to…’ Participants answered for 16 different acts of rule breaking ranging from minor acts (e.g., ‘skip doing homework for school’) to serious acts (e.g., ‘use a weapon or force to get money or things from another young person?’) (four response categories: ‘Not wrong at all’ = 0 to ‘Very wrong’ = 3). *Generalised ability to exercise self‐control* was measured using an 8 item scale asking participants ‘Do you agree with the following statements about yourself?’ relating to self‐regulation, risk taking and short‐sightedness (four response categories: ‘Strongly disagree’ = 0 to ‘Strongly agree’ = 3). At each age items were summed to create measures of personal morality and ability to exercise self‐control (with up to two items being mean‐imputed). A composite measure of crime propensity was created by standardising both measure and adding them together.^10^ For the analyses in this paper, we use a sum of crime propensity scores across all ages as a measure of crime propensity across adolescence and young adulthood. Higher scores reflect higher crime propensities.

*Criminogenic exposure* is a complex measure that utilises data from space–time budgets, community surveys and participant questionnaires. It is comprised of two measures: *time spent in criminogenic settings* qualified by a measure of *crime prone peers*. *Time spent in criminogenic settings* utilised data from the PADS+ space–time budget and the 2005 and 2012 Peterborough Community Surveys. The space–time budget gathers hourly data over 4 days in the week prior to participants' interviews covering where they were, what they were doing and who they were with, geocoded by output area to link to PCS data. Time spent in criminogenic settings was operationalised as the number of hours at each age participants spent awake outside school and work settings, unsupervised with peers, in unstructured activities (e.g., hanging around or socialising), in output areas that contain local or city centres or in output areas with poor collective efficacy (a composite measure of residents' social cohesion and informal social control described previously, extrapolated across time using change between the 2005 and 2012 PCS) (see Wikström, Oberwittler, et al. 2012; Wikström, Treiber, and Hardie 2012).

*Crime prone peers* was measured through participant questionnaires using a six item scale asking ‘Does it often happen that your peers…’ do any of six deviant acts: ‘skip school without an excuse’; ‘get drunk’; ‘sniff glue, or gas or use drugs’; ‘steal things from others or steal things from shops’; ‘destroy things that do not belong to them’; ‘beat up or are in fights with others’ (four response categories: ‘No, never’ = 0; ‘Yes, sometimes’; ‘Yes, often (every month)’ and ‘Yes, very often (every week)’ = 3). Items were summed to create a score at each age. To create a measure of criminogenic exposure, time spent in criminogenic settings and crime prone peers were standardised and added together at each age.^11^ For the analyses in this paper, we use a sum of criminogenic exposure scores across all ages as a measure of criminogenic exposure in adolescence and young adulthood. Higher scores reflect higher criminogenic exposure.
